# Effect of Ce/Zr Composition on Structure and Properties of Ce_1−x_Zr_x_O_2_ Oxides and Related Ni/Ce_1−x_Zr_x_O_2_ Catalysts for CO_2_ Methanation

**DOI:** 10.3390/nano12183207

**Published:** 2022-09-15

**Authors:** Vera P. Pakharukova, Dmitriy I. Potemkin, Vladimir N. Rogozhnikov, Olga A. Stonkus, Anna M. Gorlova, Nadezhda A. Nikitina, Evgeniy A. Suprun, Andrey S. Brayko, Vladimir A. Rogov, Pavel V. Snytnikov

**Affiliations:** 1 Boreskov Institute of Catalysis SB RAS, Pr. Lavrentieva 5, 630090 Novosibirsk, Russia; 2 Department of Natural Sciences, Novosibirsk State University, Pirogova Street 2, 630090 Novosibirsk, Russia; 3 Department of Chemistry, Moscow State University, Leninskie Gory St., 1, 119991 Moscow, Russia

**Keywords:** Ni/Ce_1−x_Zr_x_O_2_ catalysts, mixed oxides, structure, microstructure, methanation, carbon dioxide

## Abstract

Ce_1−x_Zr_x_O_2_ oxides (x = 0.1, 0.25, 0.5) prepared via the Pechini route were investigated using XRD analysis, N_2_ physisorption, TEM, and TPR in combination with density functional theory calculations. The Ni/Ce_1−x_Zr_x_O_2_ catalysts were characterized via XRD analysis, SEM-EDX, TEM-EDX, and CO chemisorption and tested in carbon dioxide methanation. The obtained Ce_1−x_Zr_x_O_2_ materials were single-phase solid solutions. The increase in Zr content intensified crystal structure strains and favored the reducibility of the Ce_1−x_Zr_x_O_2_ oxides but strongly affected their microstructure. The catalytic activity of the Ni/Ce_1−x_Zr_x_O_2_ catalysts was found to depend on the composition of the Ce_1−x_Zr_x_O_2_ supports. The detected negative effect of Zr content on the catalytic activity was attributed to the decrease in the dispersion of the Ni^0^ nanoparticles and the length of metal–support contacts due to the worsening microstructure of Ce_1−x_Zr_x_O_2_ oxides. The improvement of the redox properties of the Ce_1−x_Zr_x_O_2_ oxide supports through cation modification can be negated by changes in their microstructure and textural characteristics.

## 1. Introduction

The CO_2_ methanation process has received great interest during the last few years as a promising method of the production of synthetic natural gas as a hydrogen storage approach [1,2,3]. Nickel-based catalysts exhibit high activity and selectivity in the methanation of carbon oxides and are less expensive than systems containing noble metals [3,4,5]. Because of this, nickel catalysts are widely investigated. The impact of a support on the dispersion of nickel particles and catalytic performance of nickel catalysts is significant [6,7,8]. CeO_2_ was shown to be one of the most effective support materials [6,7,8,9,10,11,12,13,14,15,16]. Ceria works as both a support for nickel particles and a reaction promoter. The promoting effect is related to the easiness and reversibility of Ce^4+^–Ce^3+^ transition associated with appearing or healing oxygen vacancies [17]. Oxygen vacancies on the support surface were reported to participate in the activation of CO_2_ molecules [14,18,19,20,21,22].

The modification of ceria by doping with foreign cations is a well-known strategy to improve its redox properties [17]. Nickel catalysts supported on mixed Ce_1−x_Zr_x_O_2_ oxides are promising systems in terms of catalytic activity and stability [12,23,24,25,26]. Zirconium is recognized as a promoter of the reducibility and oxygen storage capacity of ceria [27,28,29]. Our previous study [26] showed that the formation of oxygen vacancies on the support surface is essential for activity of Ni/Ce_1−x_Zr_x_O_2_ catalysts in the methanation of carbon oxides. The reducibility of Ce_1−x_Zr_x_O_2_ oxides was experimentally shown to increase with the Zr content [29,30]. The regulation of the support redox properties through the variation of Zr content is of great interest. However, there have been few studies on the effect of the Ce/Zr ratio on the performance of Ni/Ce_1−x_Zr_x_O_2_ catalysts in CO_2_ methanation. Ocampo et al. [23] studied CO_2_ methanation over 5 wt.%Ni/Ce_1−x_Zr_x_O_2_ catalysts differing in Zr content (x = 0.28, 0.5, 0.86). The 5 wt.%Ni/Ce_0.5_Zr_0.5_O_2_ catalyst showed the highest activity. Nie et al. [31] studied NiO-CeO_2_-ZrO_2_ mixed oxides containing 40 wt.% Ni. The catalyst with a Ce/Zr molar ratio of 9:1 (Ce_0.9_Zr_0.1_O_2_) exhibited the best catalytic properties. Atzori et al. [32] tested NiO-CeO_2_-ZrO_2_ catalysts (30 wt.% Ni—Ce_1−x_Zr_x_O_2_) in CO_2_ and CO co-methanation. The activity was the same for the catalysts with Zr content in x = 0-0.5 and decreased at a higher content. To summarize, a correlation between the activity of Ni/Ce_1−x_Zr_x_O_2_ catalysts and the Ce/Zr ratio has not been fully understood. All the reported studies indicated that nickel catalysts based on highly doped Ce_1−x_Zr_x_O_2_ oxides (x > 0.5) are less effective. The data concerning systems based on Ce_1−x_Zr_x_O_2_ oxides with a lower Zr content (x ≤ 0.5) are somewhat controversial.

The present work aims to provide further insight into the relation between the composition of the Ce_1−x_Zr_x_O_2_ support materials and the performance of Ni/Ce_1−x_Zr_x_O_2_ catalysts in CO_2_ methanation. A series of Ce_1−x_Zr_x_O_2_ supports with different Zr contents (x = 0.1, 0.25, 0.5) were prepared via the Pechini method. The supported catalysts Ni/Ce_1−x_Zr_x_O_2_ containing 10 wt.% Ni were synthesized via the impregnation method. DFT calculations and TPR studies were employed to evaluate the impact of Zr content on the reducibility of the Ce_1−x_Zr_x_O_2_ oxides. A wide range of physical methods was used to reveal the structure features of the Ce_1−x_Zr_x_O_2_ support materials and Ni/Ce_1−x_Zr_x_O_2_ catalysts. The catalytic properties of the Ni/Ce_1−x_Zr_x_O_2_ samples were analyzed with regard to the structural features of both the support materials and catalysts.

## 2. Experimental Section

### 2.1. Computational Methods and Details

The DFT+U calculations were performed using the Vienna Ab initio Simulation Package (VASP) program [33]. The generalized gradient approximation (GGA) PBE96 functional was employed [34]. The core electrons were described by projector augmented-wave (PAW) potentials [35,36], and the valence electrons were described by a plane-wave basis set. The DFT+U method (U_eff_ = 5 eV) [37] was used to correct the strong Coulomb repulsion of cerium and DFT-D3 [38] to take into account dispersion corrections. The cutoff energy was 400 eV. A 2 × 2 × 1 Monkhorst–Pack k-point set was used for the Brillouin-zone integration.

The face-centered cubic unit cell of a fluorite-type structure (space group: Fm $\bar{3}$ m) was used as the initial geometry in the calculations [39,40]. Ce_1−x_Zr_x_O_2_ supercells (x = 0.25, 0.50, 0.75) were built by replacing Ce atoms with Zr ones (Supplementary Materials, Figure S1). Ce_1−x_Zr_x_O_2_ (100) and (111) surfaces were modeled as p (2 × 2) and p (3 × 3) slab cells. The (100) and (111) Ce_1−x_Zr_x_O_2_ supercells contained four and three layers, respectively. A vacuum space of 15° Å was set between neighboring slabs to keep the spurious interaction.

The energy of oxygen vacancy formation (E_f_) was computed as:

E_f_ = E(Ce_1−x_Zr_x_O_2−__δ_) + ½ E(O_2_) − E(Ce_1−x_Zr_x_O_2_),

where E(Ce_1−x_Zr_x_O_2−δ_) and E(Ce_1−x_Zr_x_O_2_) are the energies for surfaces with and without oxygen vacancy, and E(O_2_) is the energy of gas-phase O_2_.

### 2.2. Samples Preparation

#### 2.2.1. Synthesis of Ce_1−x_Zr_x_O_2_ Mixed Oxides

A series of Ce_1−x_Zr_x_O_2_ (x = 0.1, 0.25, 0.5) mixed oxides was prepared via the Pechini method [41]. The Ce(NO_3_)_3_*6H_2_O (99.4%) and ZrO(NO_3_)_2_*8H_2_O (99.98%) salts were used as precursors. Aqueous solutions of the salts with required Ce:Zr molar ratios (9:1; 3:1; 1:1) were prepared. Citric acid (CA) was added to the aqueous solutions at 80 °C and vigorously stirred for 30 min. The CA:metal molar ratio was 1:1. Subsequently, ethylene glycol C_2_H_4_(OH)_2_ (EG) was added. The CA:EG molar ratio was 3:2. Next, the solution was heated at 100 °C to promote the polyesterification reaction and water evaporation with the formation of a polymeric resin. The obtained solids were mechanically milled to powders and calcined at 450 °C for 8 h.

#### 2.2.2. Synthesis of Ni/Ce_1−x_Zr_x_O_2_ Catalysts

The catalysts containing 10 wt.% Ni were prepared by the impregnation of the obtained Ce_1−x_Zr_x_O_2_ oxide materials. Metal precursor Ni(CH_3_COO)_2_·4H_2_O (99.0%) and ethylene glycol (99.5%) were dissolved in the distilled water under stirring at 70°C for 20 min. Then, the support material was added into the solution. The EG:Ni molar ratio was set to 5.3. The suspension of the support and impregnating solution was stirred for 2 h at 70 °C and dried at 120 °C for 12 h in air. The dried samples were heated at a rate of 2 °C/min and calcined in air at 400 °C for 2 h. The catalysts were designated as Ni/Ce_1−x_Zr_x_O_2_, with x indicating Zr content.

### 2.3. Samples Characterization

#### 2.3.1. XRF Analysis

Elemental compositions of the catalysts were determined via X-ray fluorescent spectroscopy (XRF) using an ARL-Advant’x device (Thermo Fisher, Vienna, Austria). Measurements were carried out in a helium atmosphere using the Rh X-Ray tube. UniQuant software was used to calculate the element percentages.

#### 2.3.2. BET Surface Area Analysis

The BET specific surface areas (S_BET_, m^2^/g) of the Ce_1−x_Zr_x_O_2_ oxides were determined by N_2_-physisorption at −196 °C. The experiments were carried out using an ASAP 2400 instrument (Micrometrics, Norcross, GA, USA).

#### 2.3.3. XRD Analysis

X-ray diffraction (XRD) measurements were carried out using a D8 Advance diffractometer (Bruker, Germany) equipped with a Lynxeye linear detector. The measurements were carried out using the Cu Kα radiation (λ = 1.5418 Å) in the 2θ range of 10–83° with a step of 0.05°. XRD phase analysis was performed using the ICDD PDF-4+ database. The evaluation of substructure parameters of the Ce_1−x_Zr_x_O_2_ oxides was performed. The separation of the crystallite size (D_XRD_) and microstrain (Δd/d) effects to the line broadening was performed by means of Williamson–Hall plots [42]. From the D_XRD_ values, the crystallite surface area (S_XRD_) values were calculated assuming the crystallites were quasi-spherical:

$$S_{\mathrm{XRD}}=\frac{6000}{{\rho D}_{\mathrm{XRD}}}$$

where ρ is the theoretical density of the material (g/cm^3^).

To estimate the degree of agglomeration of the Ce_1−x_Zr_x_O_2_ crystallites, an agglomeration coefficient (ξ) was calculated on the basis of the ratio of S_BET_ and S_XRD_ values:

$$\xi =1-\frac{S_{\mathrm{BET}}}{S_{\mathrm{XRD}}}$$

The average crystallite size of nickel-containing phases was estimated by the line broadening analysis according to the Scherrer equation [43]. The Rietveld refinement was carried out using the software package Topas v.4.2 (Bruker-AXS, Karlsruhe, Germany).

#### 2.3.4. CO Pulse Chemisorption

The average sizes of metallic Ni^0^ nanoparticles in catalysts after catalytic experiments were determined using the pulse chemisorption of CO on the assumption that each surface Ni atom adsorbed one CO molecule. The measurements were carried out using a Chemosorb analyzer (Modern Laboratory Equipment, Novosibirsk, Russia). An amount of 50 mg of each sample was placed inside a U-shape quartz reactor and reduced at 350 °C in H_2_ flow (100 mL/min) for 30 min. The treated sample was subsequently cooled down to room temperature, followed by Ar purge. After that, pulses of CO were fed to the reactor (100 μL) until the amount of CO in the outlet stopped changing according to the thermal conductivity detector. The amount of chemisorbed CO was estimated. CO adsorption over the pure supports was negligible.

#### 2.3.5. TEM-EDX

Transmission electron microscopy (TEM) studies were carried out using a JEM-2200FS (JEOL, Tokyo, Japan) and a Themis Z electron microscope (Thermo Fisher Scientific, Eindhoven, The Netherlands) operated at 200 kV. Images in Scanning-TEM (STEM) mode were acquired using a high-angle annular dark field (HAADF) detector. The local elemental composition of the samples was studied using a Thermo Fisher Scientific Super-X EDX spectrometer. The samples were ground, suspended in ethanol, and placed on a copper grid coated with a holey carbon film.

#### 2.3.6. SEM-EDX

Scanning electron microscopy–energy-dispersive X-ray analysis (SEM-EDX) studies were carried out using a dual-beam scanning electron microscope, Tescan Solaris FE/SEM (Tescan, Brno, Czech Republic). The experiments were performed in secondary electron mode at an accelerating voltage of 20 kV. The microscope is equipped with an AztecLive EDX spectrometer (Oxford Instruments, High Wycombe, UK) with a Silicon Drift Detector and energy resolution of 128 eV. The cross-sections of catalyst granules in epoxy resin were prepared for the examination. The cross-sections with a given flatness of 0.25 μm were covered with a conductive carbon layer of 10–20 nm thickness.

#### 2.3.7. H_2_-TPR

The temperature-programmed reduction (TPR) via hydrogen was performed with 40–60 mg of sample in a quartz reactor using a flow setup with a thermal conductivity detector. The gas mixture containing 10 vol.% of H_2_ in Ar was fed at 40 mL/min. The rate of heating from 25 to 800 °C was 10 °C/min. The TPR curves were normalized per sample mass.

### 2.4. Tests of CO_2_ Methanation Activity

The catalytic tests were performed in a U-shaped tubular continuous-flow reactor (i.d. = 3 mm) at ambient pressure and the temperature range from 200 to 400 °C. The temperature was controlled using a K-type thermocouple placed in the middle of the catalyst bed. The feed gas contained 4 vol.% CO_2_, 16 vol.% H_2_, and Ar as balance. The catalyst load was 150 mg, 0.2–0.5 mm fraction, and the flow rate—75 mL/min. Prior to the experiment, each catalyst was reduced in 10 vol.% H_2_ in Ar flow (50 mL/min) at 400 °C for 1 h. The compositions of the inlet and outlet gas mixtures were determined using a gas chromatograph, KHROMOS-1000 (Khromos, Dzerzhinsk, Russia), equipped with a thermal conductivity detector (CaA molecular sieves column) and flame ionization detector (Porapak Q column) with a methanator characterized by sensitivity to CO, CH_4_, and CO_2_ of ~1 ppm. The separation of CO, CH_4_, and CO_2_ on the column followed by the methanation of carbon oxides allowed the flame ionization detector to be used to analyze their concentration. The equilibrium compositions were calculated using equilibrium software HSC 7.0. It was assumed that equilibrium mixtures only contained gaseous substances (CH_4_, CO, CO_2_, H_2_, and H_2_O), i.e., no carbon deposition processes were taken into account. The catalytic properties of Ni/Ce_1−x_Zr_x_O_2_ catalysts were also compared with those of industrial catalyst NIAP-07-05 for the methanation of carbon oxides, which contains 38 wt.% NiO, 12 wt.% Cr_2_O_3_, and 50 wt.% Al_2_O_3_ (further denoted as 38Ni−Cr−Al). The catalyst has a Ni^0^-specific surface area of 3.3 m^2^/g in a reduced state [26].

## 3. Results and Discussion

### 3.1. Calculation Results

According to DFT+U calculations, the parameter of the Ce_1−x_Zr_x_O_2_ lattice decreases with an increase in Zr content (Table 1) due to a smaller radius of the Zr^4+^ cation (0.84 Å) compared to the Ce^4+^ cation (0.97 Å). Doping CeO_2_ with Zr leads to a slight decrease in Ce–O distances. The calculated Ce–O and Zr–O distances are in the range of 2.314–2.375 Å and 2.205–2.258 Å, respectively.

The calculated values of the surface energy are presented in Table 1. The Ce_1−x_Zr_x_O_2_ (111) surface is more stable than the (100) surface. The CeO_2_ (111) surface is known to be the most stable surface among the low-index surfaces (100), (110), and (111) [44,45]. Zirconium incorporation slightly increases the surface energy at 0 < x < 0.5, while a sharp increase in the energy is observed at x > 0.5.

The calculated energies of oxygen vacancy formation in Ce_1−x_Zr_x_O_2_ are also listed in Table 1. The E_f_ values decrease considerably in the range of compositions 0 < x < 0.5, while at x > 0.5, the E_f_ values increase. The Ce_0.5_Zr_0.5_O_2_ surfaces are characterized by the lowest energies of oxygen formation. It was found that Zr incorporation facilitates the generation of oxygen vacancy. The obtained result agrees with data of interatomic potential simulations reported by G. Balducci et al. [46,47], which showed that Ce^4+^/Ce^3+^ reduction energy is reduced even by small amounts of zirconium incorporated into ceria. Based on DFT+U calculations, Yang et al. [48,49] also found that zirconium addition leads to lowering energy of the oxygen vacancy formation. The structure distortion induced by Zr cations can be responsible for the decrease in the reduction energy. It was assumed that the smaller Zr^4+^ cations counterbalance steric strains arising at formation Ce^3+^ cations, which are larger than Ce^4+^ cations [46,49].

### 3.2. Experimental Results

#### 3.2.1. Ce_1−x_Zr_x_O_2_ Support Materials

**Figure 1:**
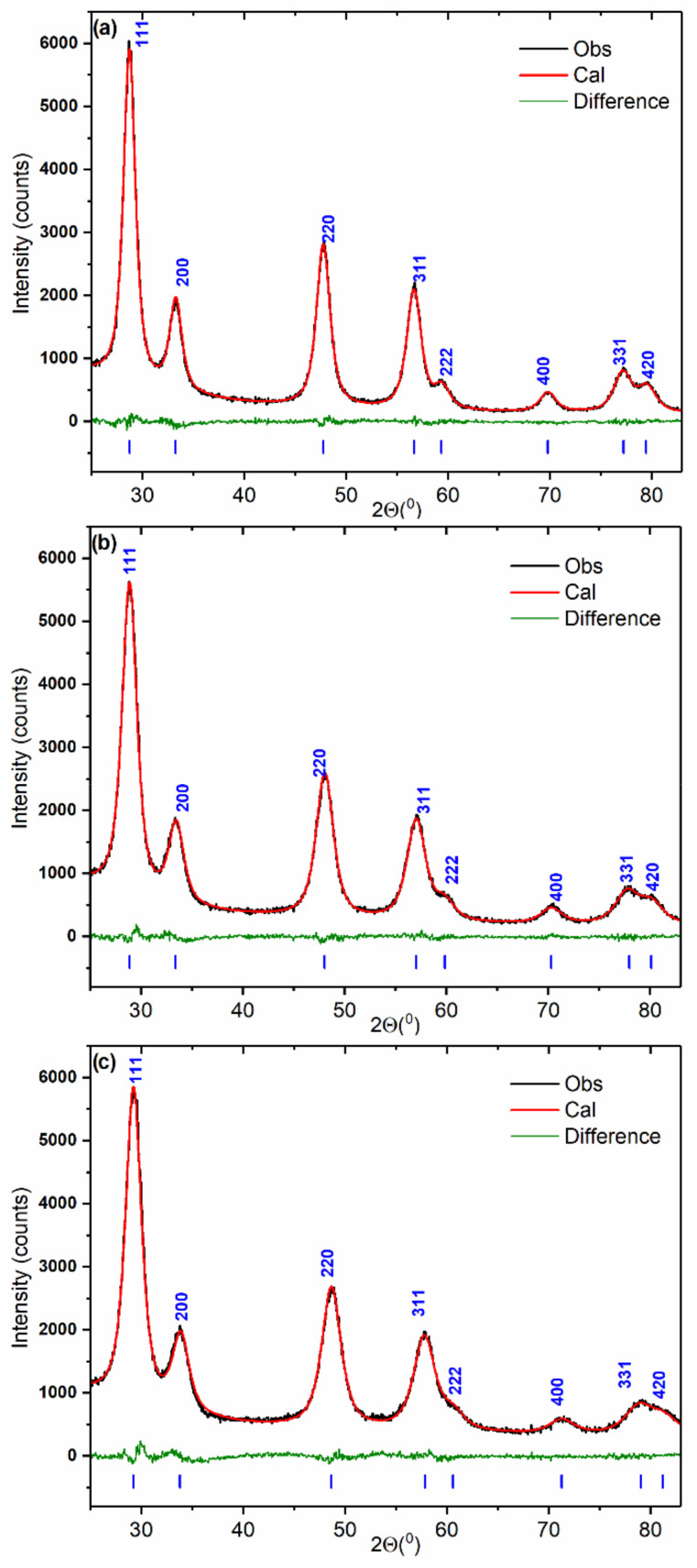
XRD patterns fitted using the Rietveld refinement method for Ce_0.9_Zr_0.1_O_2_ (**a**), Ce_0.75_Zr_0.25_O_2_ (**b**), and Ce_0.5_Zr_0.5_O_2_ (**c**) mixed oxides.

The powder XRD patterns of the Ce_1−x_Zr_x_O_2_ samples are shown in Figure 1. Only Bragg peaks related to oxide with a fluorite-type cubic crystal structure (S.G. Fm $\bar{3}$ m) are observed.

Table 2 lists data on the structural parameters obtained by Rietveld refinement. The lattice parameters of all the oxides are lower than the value characteristic of CeO_2_ (a = 5.411 Å, PDF# 00-028-0753). The lattice shrinkage indicates the formation of substitutional solid solution Ce_1−x_Zr_x_O_2_. The gradual decrease in the lattice parameter with the increase in Zr content is observed. The composition of the Ce_1−x_Zr_x_O_2_ solid solutions was evaluated with use of Vegard’s rule. The linear dependence of the lattice parameter on the Zr content is provided elsewhere [26]. Estimated values of zirconium content coincide with those set at the synthesis (Table 2). This implies that obtained materials are single-phase Ce_1−x_Zr_x_O_2_ solid solutions. As can be seen from Table 2, the isotropic temperature factors of atoms increase with Zr content. This suggests the increase in crystal lattice distortion resulted from Zr incorporation.

Microstrain analysis also indicates the intensification of structure deformation with an increasing Zr concentration in Ce_1−x_Zr_x_O_2_ oxides (Table 3). The microstrain value for the Ce_0.5_Zr_0.5_O_2_ sample is roughly two times higher than that for the Ce_0.9_Zr_0.1_O_2_ sample. A difference in the radii of Ce^4+^ and Zr^4+^ cations is the main reason for the crystal lattice distortion. As mentioned above, the structure strains induced by Zr doping can be responsible for the improved reducibility of the Ce_1−x_Zr_x_O_2_ mixed oxides.

**Figure 2:**
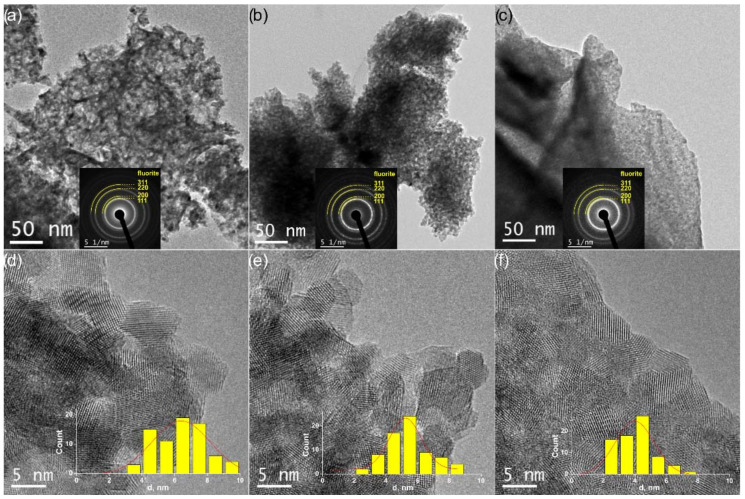
TEM data for Ce_0.9_Zr_0.1_O_2_ (**a**,**d**), Ce_0.75_Zr_0.25_O_2_ (**b**,**e**), and Ce_0.5_Zr_0.5_O_2_ (**c**,**f**) catalysts: (**a**–**c**) TEM images and SAED patterns in insets with indication of fluorite phase rings; (**d**–**f**) HRTEM images and particle size distribution histograms.

All the oxides are highly dispersed; the D_XRD_ are in the range of 5–7 nm. However, quite low S_BET_ values were obtained. Calculated S_XRD_ values significantly exceed S_BET_ ones (Table 3). Such a difference between S_XRD_ and S_BET_ values can be explained by the agglomeration of primary crystallites. The analysis of estimated agglomeration coefficients (Table 3) showed that an increase in Zr content in the Ce_1−x_Zr_x_O_2_ oxides is accompanied with an increase in the agglomeration of crystallites. TEM data confirmed these results. The TEM images shown in Figure 2a–c demonstrate the loose agglomeration of crystallites in the low-doped Ce_0.9_Zr_0.1_O_2_ sample and the significantly denser agglomeration of crystallites in the high-doped Ce_0.5_Zr_0.5_O_2_ sample. In the HRTEM images (Figure 2d–f), the individual Ce_1−x_Zr_x_O_2_ crystallites are distinguishable. The mean size of Ce_1−x_Zr_x_O_2_ crystallites (Table 3, d_HRTEM_) was determined from particle size distribution histograms shown in insets in Figure 2d–f. There is a clear tendency for the Ce_1−x_Zr_x_O_2_ crystallite size to decrease as the Zr content increases in accordance with the XRD results.

**Figure 3:**
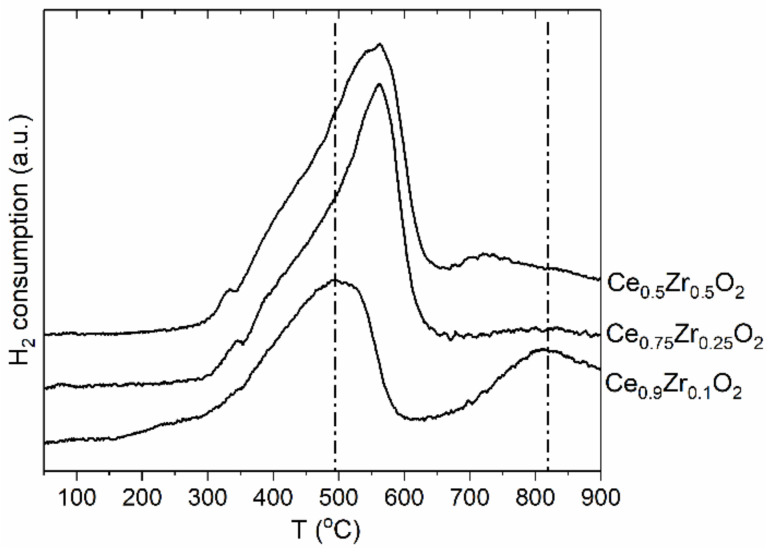
The H_2_-TPR profiles of the Ce_1−x_Zr_x_O_2_ samples.

The reduction behavior of the Ce_1−x_Zr_x_O_2_ oxides was studied. The H_2_-TPR profiles are reported in Figure 3. The H_2_-TPR profile of the low-doped Ce_0.9_Zr_0.1_O_2_ oxide exhibits two broad peaks at 500 °C and 820 °C. Such a two-peak pattern is characteristic of CeO_2_ oxide and reflects the stepwise reduction in the surface and bulk [50,51]. The TPR profiles of the Ce_0.75_Zr_0.25_O_2_ and Ce_0.5_Zr_0.5_O_2_ oxides exhibit asymmetric peaks with significantly increased intensities. The second high-temperature reduction peak is not observed. These data indicate that surface and bulk reduction processes occur almost simultaneously. The observed intensive asymmetric peak reflects the co-reduction in surface and bulk. This suggests the improvement of the reducibility of the mixed Ce_1−x_Zr_x_O_2_ oxides with an increase in Zr content in full agreement with previous TPR studies of Ce_1−x_Zr_x_O_2_ oxides [28,29,30].

#### 3.2.2. Ni/Ce_1−x_Zr_x_O_2_ Catalysts

**Figure 4:**
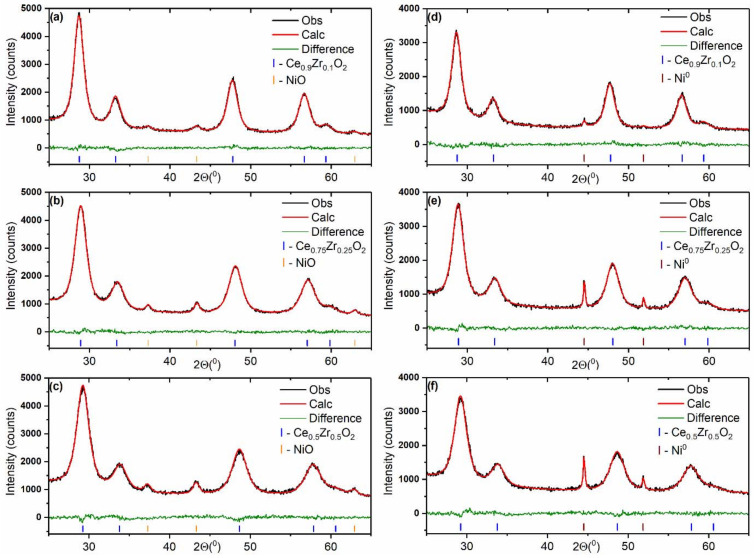
Fragments of XRD patterns fitted using the Rietveld refinement method for Ni/Ce_0.9_Zr_0.1_O_2_ (**a**,**d**), Ni/Ce_0.75_Zr_0.25_O_2_ (**b**,**e**), and Ni/Ce_0.5_Zr_0.5_O_2_ (**c**,**f**) catalysts before (**a**–**c**) and after (**d**–**f**) the catalytic reaction.

Figure 4 shows XRD patterns of the Ni/Ce_1−x_Zr_x_O_2_ catalysts. The broad peaks from the oxide NiO phase (PDF#00-047-1049) are detected in the XRD patterns of the as-prepared catalysts, while the narrow peaks from the metallic Ni^0^ phase (PDF #00-004-0085) are observed in the XRD patterns of the catalysts aged under reductive conditions of CO_2_ methanation.

Table 4 summarizes the average size characteristics of nickel species in the as-prepared and used Ni/Ce_1−x_Zr_x_O_2_ catalysts according to the XRD and CO chemisorption data. The Ni/Ce_0.9_Zr_0.1_O_2_ catalyst is characterized by a higher dispersion of initial NiO crystallites as well as Ni^0^ crystallites formed upon reduction. Reaction conditions provoke the sintering of nickel species. The average size of Ni^0^ crystallites in the aged catalysts is larger than the size of NiO crystallites in the as-prepared catalysts. The Ni/Ce_0.9_Zr_0.1_O_2_ catalyst is characterized by a higher resistance of Ni^0^ crystallites to sintering. The average size of Ni^0^ crystallites in the Ni/Ce_0.9_Zr_0.1_O_2_ catalyst is about two times smaller than in other catalysts. CO chemisorption results (Table 4) confirmed that the Ni/Ce_0.9_Zr_0.1_O_2_ catalyst contains Ni^0^ particles of the highest dispersion. The determined sizes of Ni^0^ nanoparticles in the aged catalysts are in the increasing order of Ni/Ce_0.9_Zr_0.1_O_2_ < Ni/Ce_0.75_Zr_0.25_O_2_ < Ni/Ce_0.5_Zr_0.5_O_2_. The chemisorption method is considerably more sensitive to ultrafine particles compared to XRD analysis. The observed differences in sizes measured via the chemisorption and XRD techniques suggest that highly dispersed particles of metallic Ni^0^, undetectable via XRD, are present in the catalysts.

The comparison of values of Ni^0^ content determined from the Rietveld refinement of XRD data and XRF analysis (Supplementary material, Table S1) confirmed that a part of the loaded nickel in the catalysts is not detected via XRD analysis. The fraction of XRD-undetectable nickel species in the catalysts decreases in the sequence: Ni/Ce_0.9_Zr_0.1_O_2_ > Ni/Ce_0.75_Zr_0.25_O_2_ > Ni/Ce_0.5_Zr_0.5_O_2_.

**Figure 5:**
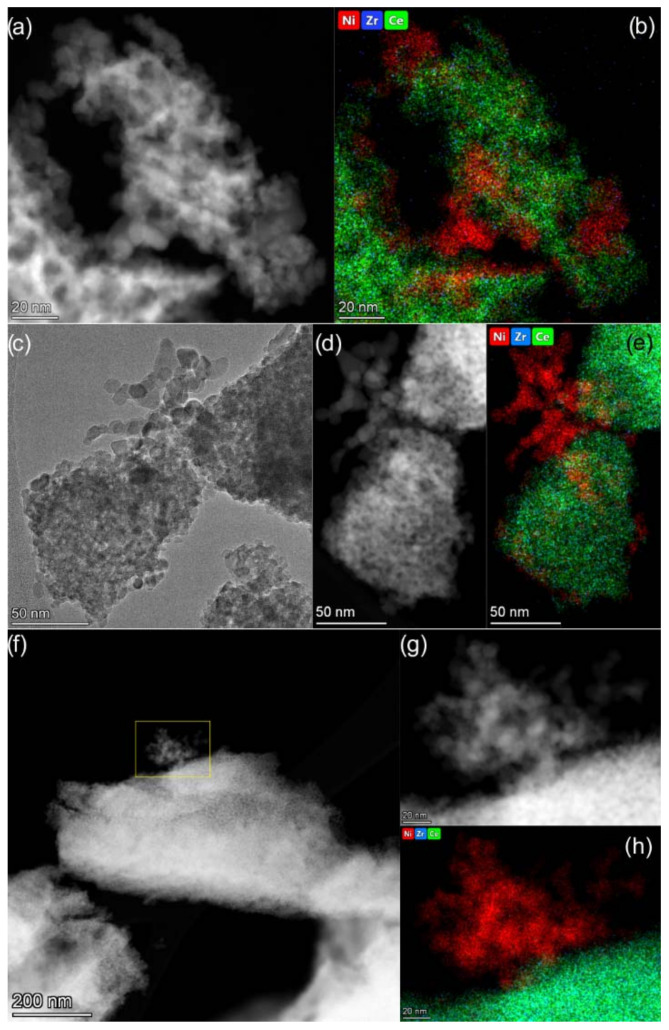
TEM data for Ni/Ce_0.9_Zr_0.1_O_2_ (**a**,**b**), Ni/Ce_0.75_Zr_0.25_O_2_ (**c**–**e**), and Ni/Ce_0.5_Zr_0.5_O_2_ (**f**–**h**) catalysts: TEM (**c**) and HAADF STEM (**a**,**d**,**f**,**g**) images and corresponding EDX mapping patterns (**b**,**e**,**h**) showing distribution of Ni (red), Zr (blue), and Ce (green). The maps are presented in background-corrected intensities.

The HAADF STEM images and EDX mapping patterns are presented in Figure 5. The analysis of EDX data revealed that the spatial distribution of Ce and Zr in the catalysts is homogeneous. No Ce-rich or Zr-rich areas are observed. This confirms that Ce_1−x_Zr_x_O_2_ oxides used as supports are single-phase substitutional solid solutions. The analysis of Ni distribution via EDX allows us to see Ni-rich nanoparticles of 5−10 nm in size in the catalysts. These particles in all the studied samples were shown to be NiO particles (Supplementary material Figure S2). Individual NiO nanoparticles in contact with support particles and ones assembled into large aggregates are observed. The Ni/Ce_1−x_Zr_x_O_2_ catalysts were found to differ in the amount of non-agglomerated NiO nanoparticles and the possibility of their fixation as single particles. Thus, a large number of single NiO particles being in contact with support particles are observed in the images of the Ni/Ce_0.9_Zr_0.1_O_2_ catalyst (Figure 5b). More developed contacts between NiO particles and Ce_0.9_Zr_0.1_O_2_ support are likely responsible for the higher resistance of nickel particles to sintering under conditions of CO_2_ methanation. In the case of the Ni/Ce_0.5_Zr_0.5_O_2_ catalyst, agglomerated NiO nanoparticles with slight contact with the support are observed (Figure 5h). The Ni particles undergo reduction and sintering during the reaction, as revealed by XRD analysis. However, the highly dispersed particles fixed on the support are retained (Supplementary material Figure S3).

**Figure 6:**
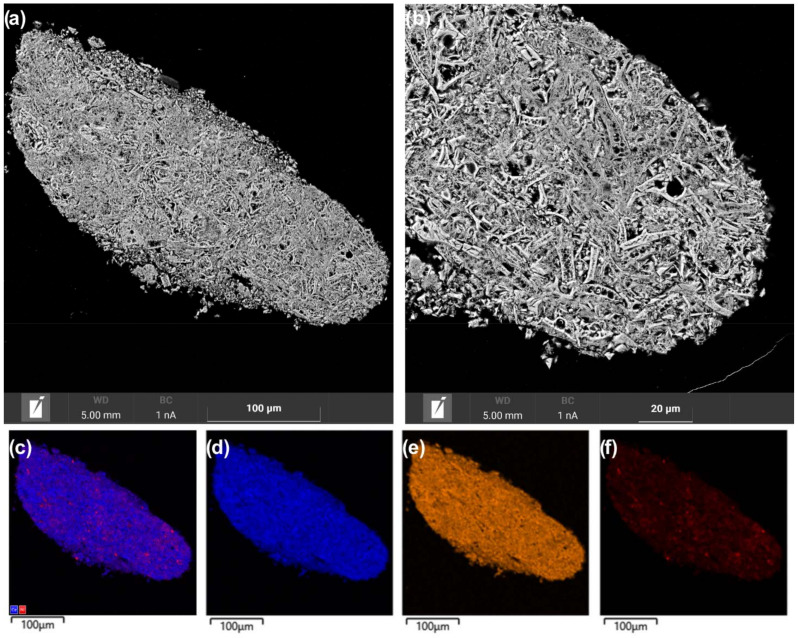
SEM images (**a**,**b**) and EDX mapping patterns (**c**–**f**) for nickel (red), zirconium (orange), and cerium (blue) of cross-section area of the Ni/Ce_0.5_Zr_0.5_O_2_ catalyst.

The observed differences in the dispersion of nickel species in the catalysts seem to be related to effect of the microstructure of the Ce_1−x_Zr_x_O_2_ oxides on the formation of supported nanoparticles. As noted above, the Ce_1−x_Zr_x_O_2_ support materials differed in particle organization and the agglomeration of crystallites. The microstructure features of the catalysts were additionally studied via SEM coupled with EDX analysis. SEM analysis showed quite different internal organization and porous structure in grains of the Ni/Ce_0.9_Zr_0.1_O_2_ and Ni/Ce_0.5_Zr_0.5_O_2_ catalysts. In the Ni/Ce_0.5_Zr_0.5_O_2_ catalyst, coarse elongated support particles are aggregated with the formation of large, unevenly distributed voids (Figure 6a,b). Large NiO particles are visualized in the big voids, while the main part of the support material is weakly covered with nickel compounds according to EDX mapping (Figure 6c,f).

**Figure 7:**
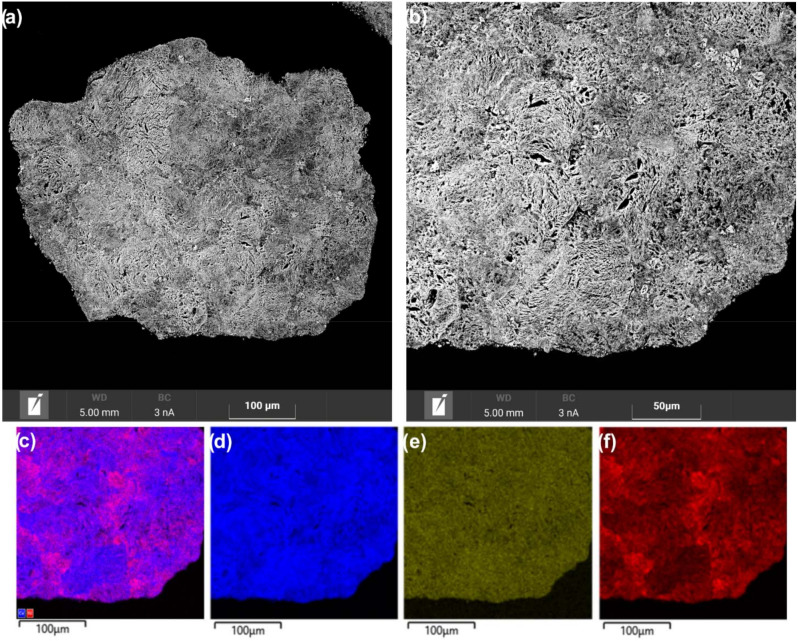
SEM images (**a**,**b**) and EDX mapping patterns (**c**–**f**) for nickel (red), zirconium (green), and cerium (blue) of cross-section area of the Ni/Ce_0.9_Zr_0.1_O_2_ catalyst.

In the Ni/Ce_0.9_Zr_0.1_O_2_ catalyst, aggregates of smaller and thinner support particles have a more uniform distribution of narrow interparticle voids (Figure 7a,b). EDX mapping showed the more homogeneous distribution of nickel over the Ce_0.9_Zr_0.1_O_2_ support (Figure 7c,f) than over the Ce_0.5_Zr_0.5_O_2_ support (Figure 6c,f). It appears that a network of narrow channels provided a more uniform distribution of the nickel compounds over the support surface in the impregnation step. As a result, the support is effectively covered with nickel compounds and there is no pronounced gradient in the size of formed NiO particles. Thus, the SEM-EDX results allowed one to explain the observed differences in Ni/Ce_1−x_Zr_x_O_2_ catalysts in the dispersion of nickel species. The microstructure features of the Ce_1−x_Zr_x_O_2_ oxides affected the dispersion of nickel compounds as well as their spatial distribution over the supports. The microstructure of the Ce_0.9_Zr_0.1_O_2_ support is more beneficial for the uniform distribution of the nickel compounds without their segregation.

The formation of larger particles in the Ce_0.5_Zr_0.5_O_2_ oxide seems to be caused by the considerable aggregation of the constituent crystallites (Table 3, Figure 2). On the one hand, greater aggregation is likely related to the higher dispersion of crystallites. Ultrafine crystallites assemble to lower the surface energy [52,53]. On the other hand, differences in the microstructure can be related with specifics of the formation of Ce_1−x_Zr_x_O_2_ oxides at their preparation via the Pechini route. Thus, the intensity of combustion processes when burning the organic polymer matrix depends on the relative contents of Ce and Zr atoms. During the preparation of Ce_0.9_Zr_0.1_O_2_ oxide, a higher content of easily oxidized Ce cations intensifies combustion processes. The explosive formation of gaseous products favors the formation of loose particles with the low agglomeration of crystallites.

**Figure 8:**
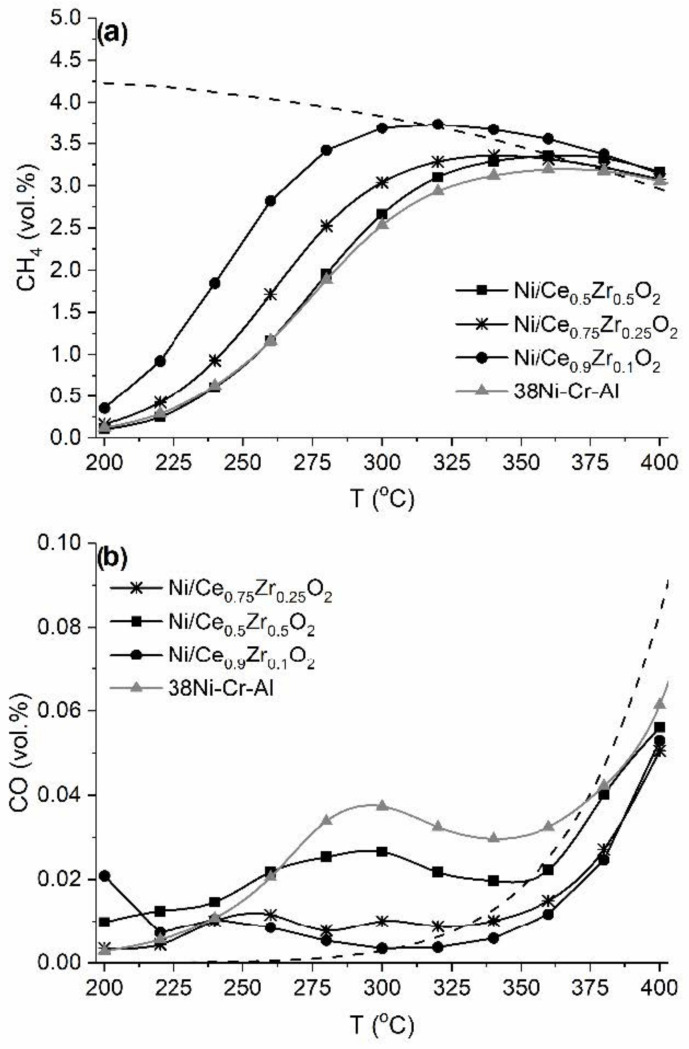
Temperature dependences of the outlet concentrations of CH_4_ (**a**) and CO (**b**) during CO_2_ methanation over the Ni/Ce_1−x_Zr_x_O_2_ catalysts and 38Ni-Cr-Al catalyst. The dashed lines correspond to the calculated equilibrium concentrations.

The catalytic performance of the Ni/Ce_1−x_Zr_x_O_2_ catalysts in CO_2_ methanation was studied. Figure 8 shows light-off curves for the CO_2_ methanation. For all the catalysts, the CH_4_ concentration increases with temperature, reaches a maximum, and then decreases coinciding with the equilibrium values. All the Ni/Ce_1−x_Zr_x_O_2_ catalysts had comparable or higher activity in comparison with the industrial 38Ni-Cr-Al catalyst containing a significantly higher amount of nickel (38 wt.% vs. 10 wt.%). In a previous work, we reported that the synergism of redox properties of the Ni/Ce_1−x_Zr_x_O_2_ system enhances the catalytic performance in the methanation of carbon oxides [26].

The Ni/Ce_0.9_Zr_0.1_O_2_ catalyst exhibited the highest catalytic activity. The CO_2_ half-conversion temperature (T_50_) was 244, 266, and 280°C for Ni/Ce_0.9_Zr_0.1_O_2_, Ni/Ce_0.75_Zr_0.25_O_2_, and Ni/Ce_0.5_Zr_0.5_O_2_, respectively. The observed decrease in activity in the sequence Ni/Ce_0.9_Zr_0.1_O_2_ >> Ni/Ce_0.75_Zr_0.25_O_2_ > Ni/Ce_0.5_Zr_0.5_O_2_ correlates with a decrease in Ni^0^-specific surface area: 6 >> 2.9 > 2 m_Ni_^2^/g_cat_. The revealed higher dispersion of Ni^0^ particles and more developed contacts between them and support surface can explain the higher catalytic activity of the Ni/Ce_0.9_Zr_0.1_O_2_ catalyst. As was shown above, the Ni/Ce_0.9_Zr_0.1_O_2_ catalyst contained Ni^0^ nanoparticles with d_Ni_^chem^ sizes of 11.2 nm, and the least active Ni/Ce_0.5_Zr_0.5_O_2_ catalyst contained Ni^0^ nanoparticles with d_Ni_^chem^ sizes of 33.7 nm (Table 4). It was also revealed that the most active Ni/Ce_0.9_Zr_0.1_O_2_ catalyst is characterized by more developed contacts between nickel particles and the support (Figure 5b). It is well accepted that high Ni^0^ dispersion and a developed metal–support interface area are highly important for the catalytic activity for CO_2_ methanation [6,8,23,54,55].

It was recently suggested that zirconium addition impacts the dispersion of nickel nanoparticles in Ni/Ce_1−x_Zr_x_O_2_ catalysts as well as the degree of metal–support interaction [23,31]. The results of this study imply that the cation composition of the Ce_1−x_Zr_x_O_2_ oxides can affect their microstructure and texture features and, consequently, the dispersion of supported nickel nanoparticles. This influence is likely related to the synthesis technique used. The Ce_1−x_Zr_x_O_2_ oxides under study were prepared via the Pechini method. A similar effect was observed by Iglesias et al. [24] in the study of Ni/Ce_1−x_Zr_x_O_2_ catalysts with supports prepared using the coprecipitation method. It was shown that an increase in Zr content diminishes the specific surface area of the Ce_1−x_Zr_x_O_2_ supports with a decrease in the dispersion of supported Ni^0^ nanoparticles. It was also reported that the increase in Zr content reduces the specific surface area of the Ce_1−x_Zr_x_O_2_ oxides prepared via the sol–gel technique [56] and the citrate complexation route [57].

## 4. Conclusions

In this study, Ce_1−x_Zr_x_O_2_ mixed oxides with different compositions were prepared using the Pechini method and used as the supports for Ni/Ce_1−x_Zr_x_O_2_ catalysts. It was demonstrated that an increase in Zr content enhances the distortion of the crystal structure of Ce_1−x_Zr_x_O_2_ oxides and leads to improvements in their redox properties. It was also found that microstructure features of Ce_1−x_Zr_x_O_2_ oxides change with cation composition.

The effect of the Ce_1−x_Zr_x_O_2_ composition on the structure and catalytic activity of the Ni/Ce_1−x_Zr_x_O_2_ catalysts in CO_2_ methanation was investigated. The activity of the Ni/Ce_1−x_Zr_x_O_2_ catalysts decreased in the order: Ni/Ce_0.9_Zr_0.1_O_2_ >> Ni/Ce_0.75_Zr_0.25_O_2_ > Ni/Ce_0.5_Zr_0.5_O_2_. The drop in the activity correlated with the decrease in the dispersion of metallic Ni^0^ nanoparticles. It was revealed that differences in the microstructural characteristics of the Ce_1−x_Zr_x_O_2_ supports are responsible for differences in the dispersion of supported Ni^0^ nanoparticles and the length of the metal–support interface.

It was shown that improving the redox properties of Ce_1−x_Zr_x_O_2_ oxides, which are important for catalysis, through cation modification can be counterbalanced by worsening their microstructure characteristics, which determine the dispersion of supported Ni^0^ nanoparticles.

## Figures and Tables

**Table 1:** Calculated geometric characteristics, energies of oxygen vacancy formation (E_f_), and surface energies (E_surf_) for Ce_1−x_Zr_x_O_2_.

Ce_1−x_Zr_x_O_2_ Composition	Lattice Parameter (Å)	r_Ce-O_ (Å)	r_Zr-O_ (Å)	E_surf_ (J/m^2^)		E_f_ (eV)	
				(100)	(111)	(100)	(111)
CeO_2_	5.46	2.375	-	1.76	0.72	2.03	2.71
Ce_0.75_Zr_0.25_O_2_	5.38	2.347	2.258	1.79	0.74	1.45	2.23
Ce_0.5_Zr_0.5_O_2_	5.26	2.326	2.242	1.81	0.75	1.22	1.95
Ce_0.25_Zr_0.75_O_2_	5.17	2.314	2.220	1.85	0.79	1.97	2.54
ZrO_2_	5.08	-	2.205	1.94	0.89	4.26	5.08

**Table 2:** Structural parameters of Ce_1−x_Zr_x_O_2_ oxides determined from XRD data.

Sample Ce_1−x_Zr_x_O_2_	Lattice Parameter (Å)	Estimated Zirconium Content *x*	B_Me_ (Å^−1^) *	B_O_ (Å^−1^) *	R_wp_ **	χ^2^ **
Ce_0.9_Zr_0.1_O_2_	5.388 (1)	0.10	0.06	0.17	3.62	0.97
Ce_0.75_Zr_0.25_O_2_	5.353 (1)	0.25	0.13	0.29	4.07	1.22
Ce_0.5_Zr_0.5_O_2_	5.290 (1)	0.51	0.45	1.20	3.92	1.58

* Isotropic temperature factors were refined on an assumption of Ce_1−x_Zr_x_O_2_ composition set at the synthesis. The factors of Ce and Zr atoms were constrained to be equal (B_Me_). ** Rietveld analysis agreement indices.

**Table 3:** Average crystallite sizes and microstrain values according to XRD data, specific surface areas calculated from XRD-derived crystallite sizes and determined by BET method, agglomeration coefficients, and average crystallite sizes according to HRTEM data.

Sample	Δd/d	D_XRD_ (nm)	S_XRD_ (m^2^/g)	S_BET_ (m^2^/g)	Agglomeration Coefficient ξ	d_HRTEM_ (nm)
Ce_0.9_Zr_0.1_O_2_	(2.6 ± 0.2) × 10^−3^	7.0	120	71	0.41	6.4
Ce_0.75_Zr_0.25_O_2_	(4.5 ± 0.2) × 10^−3^	5.5	157	83	0.47	5.5
Ce_0.5_Zr_0.5_O_2_	(6.9 ± 0.2) × 10^−3^	5.0	180	53	0.70	4.1

**Table 4:** Average size characteristics of nickel species in the as-prepared and used Ni/Ce_1−x_Zr_x_O_2_ catalysts according to the XRD and CO chemisorption data.

Sample	As-Prepared Catalysts	Aged Catalysts		
	d_NiO_^XRD^ (nm)	d_Ni_^XRD^ (nm)	d_Ni_^chem^ (nm)	S_Ni_^chem^ (m^2^/g_cat_)
Ni/Ce_0.9_Zr_0.1_O_2_	8.5(5)	20.0(5)	11.2	6
Ni/Ce_0.75_Zr_0.25_O_2_	12.0(5)	54.0(5)	23.2	2.9
Ni/Ce_0.5_Zr_0.5_O_2_	11.0(5)	53.0(5)	33.7	2

## Data Availability

Not applicable.

## Supplementary Materials

The following supporting information can be downloaded at: https://www.mdpi.com/article/10.3390/nano12183207/s1, Figure S1: The models of fluorite Ce_1−x_Zr_x_O_2_ unit cells: (a) x = 0, (b) x = 0.25, (c) x = 0.5, (d) x = 0.75, (e) x = 1; Figure S2: TEM image of as-prepared Ni/Ce_0.9_Zr_0.1_O_2_ catalyst (a), electron diffraction pattern (b); Figure S3: HAADF-STEM images and corresponding EDX-mapping patterns for aged Ni/Ce_0.9_Zr_0.1_O_2_ (a,b) Ni/Ce_0.5_Zr_0.5_O_2_ (c,d) catalysts. Table S1: Quantities of nickel compounds in the used Ni/Ce_1−x_Zr_x_O_2_ catalysts according to XRD phase analysis and XRF analysis.
