# Supplementary material for: Effect of Ce/Zr Composition on Structure and Properties of Ce1−xZrxO2 Oxides and Related Ni/Ce1−xZrxO2 Catalysts for CO2 Methanation
Source: Nanomaterials (Basel). 2022 Sep 15;12(18):3207. doi: 10.3390/nano12183207 (PMC9500888; doi:10.3390/nano12183207)
Supplement: Supplementary file 1 [file nanomaterials-12-03207-s001.zip › nanomaterials-1908588-supplementary.pdf]

nanomaterials

Supplementary Materials

Effect of Ce/Zr Composition on Structure and Properties of $Ce_{1-x}Zr_xO_2$ Oxides and Related Ni/$Ce_{1-x}Zr_xO_2$ Catalysts for CO_2 Methanation

Vera P. Pakharukova [1,*], Dmitriy I. Potemkin [1], Vladimir N. Rogozhnikov [1], Olga A. Stonkus [1], Anna M. Gorlova [1,2], Nadezhda A. Nikitina [1,3], Evgeniy A. Suprun [1], Andrey S. Brayko [1], Vladimir A. Rogov [1] and Pavel V. Snytnikov [1]

1 Boreskov Institute of Catalysis SB RAS, Pr. Lavrentieva 5, 630090 Novosibirsk, Russia
2 Department of Natural Sciences, Novosibirsk State University, Pirogova Street 2, 630090 Novosibirsk, Russia
3 Department of Chemistry, Moscow State University, Leninskie Gory St., 1, 119991 Moscow, Russia
* Correspondence: verapakh@catalysis.ru; Tel.: +7-383-326-9597; Fax: +7-383-330-8056

DFT+U Calculations

The face-centered cubic unit cell of fluorite type structure (space group: Fm $\bar{3}$ m) was used as the initial geometry in the calculations. $Ce_{1-x}Zr_xO_2$ supercells (x = 0.25, 0.50, 0.75) were built by replacing cerium atoms with zirconium ones (Figure S1).

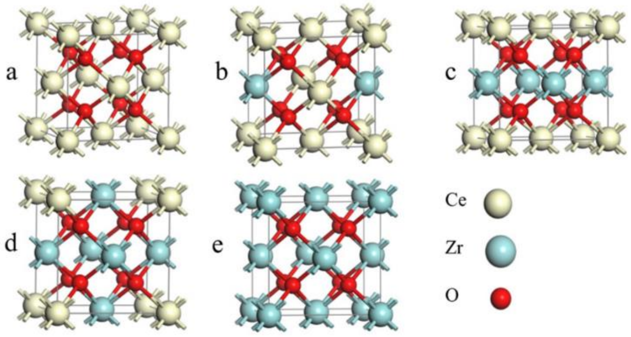

Figure S1. The models of fluorite $Ce_{1-x}Zr_xO_2$ unit cells: (**a**) x = 0, (**b**) x = 0.25, (**c**) x = 0.5, (**d**) x = 0.75, (**e**) x = 1.

XRD Quantitative Analysis of Ni/$Ce_{1-x}Zr_xO_2$ Catalysts Aged Under Reductive Conditions of CO_2 Methanation

The Rietveld refinement for quantitative analysis was carried out. The obtained data on amounts of detected crystalline Ni^0 phase were compared with XRF data on nickel loading in the catalysts (Table S1). It is seen that part of the loaded nickel in the catalysts is not detected by XRD analysis as crystalline phases. The fraction of undetectable nickel species in the catalysts decreases in order of Ni/$Ce_{0.9}Zr_{0.1}O_2$ > Ni/$Ce_{0.75}Zr_{0.25}O_2$ > Ni/$Ce_{0.5}Zr_{0.5}O_2$.

Nanomaterials **2022**, *12*, 3207. https://doi.org/10.3390/nano12183207 www.mdpi.com/journal/nanomaterials

Table S1. Quantities of nickel compounds in the used Ni/$Ce_{1-x}Zr_xO_2$ catalysts according to XRD phase analysis and XRF analysis.

Sample	XRD Quantitative Phase Analysis by Rietveld Refinement			XRF Analysis	XRD Undetected ** (%)
	R_{wp} *	χ^2 *	Quantity of Ni^0 Phase (wt.%)	Ni Loading (wt.%)	
Ni/$Ce_{0.9}Zr_{0.1}O_2$	4.01	1.03	4.5(3)	10.5(1)	57
Ni/$Ce_{0.75}Zr_{0.25}O_2$	3.23	0.94	7.2(2)	10.1(1)	28
Ni/$Ce_{0.5}Zr_{0.5}O_2$	3.18	1.01	8.9(2)	10.9(1)	18

* Rietveld analysis agreement indices. ** Fraction of nickel compounds undetectable by XRD phase analysis.

TEM Study of Ni/$Ce_{1-x}Zr_xO_2$ Catalysts

The observed nickel containing particles in all the as-prepared Ni/$Ce_{1-x}Zr_xO_2$ catalysts were found to be NiO particles. Interplanar distances measured from selected area diffraction correspond to the NiO phase, as demonstrated by the example of the Ni/$Ce_{0.9}Zr_{0.1}O_2$ catalyst (Figure S2).

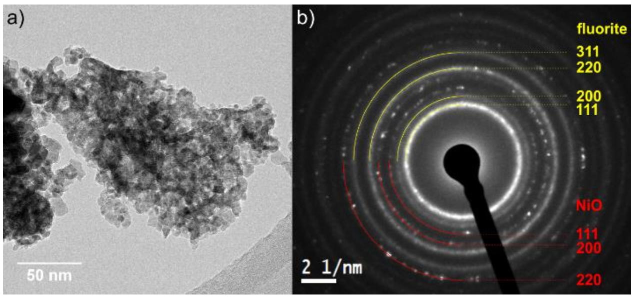

Figure S2. TEM image of as-prepared Ni/$Ce_{0.9}Zr_{0.1}O_2$ catalyst (**a**), electron diffraction pattern (**b**).

HAADF-STEM studies with EDX-mapping (Figure S3) of the spent catalysts indicated that highly dispersed nickel particles being in contact with support are preserved after catalytic reaction.

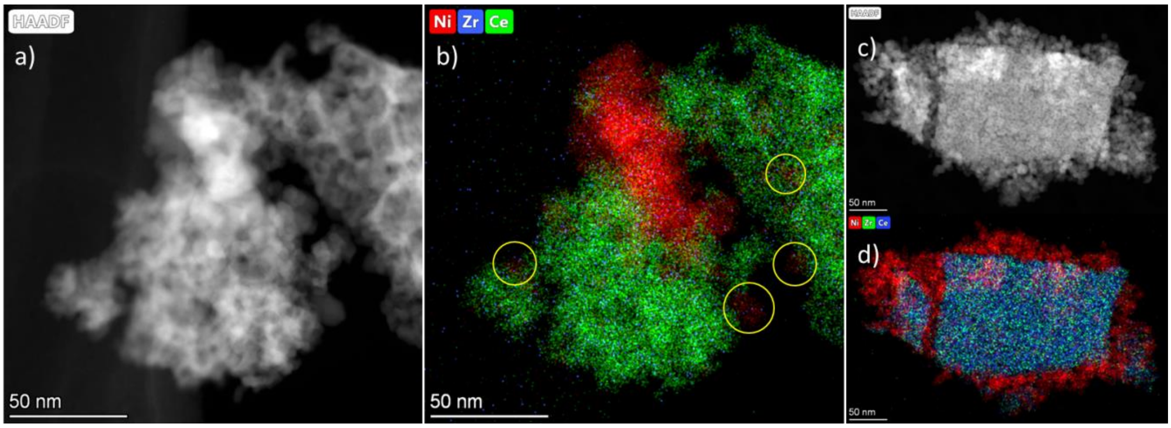

Figure S3. HAADF-STEM images and corresponding EDX-mapping patterns for aged Ni/$Ce_{0.9}Zr_{0.1}O_2$ (**a**,**b**) Ni/$Ce_{0.5}Zr_{0.5}O_2$ (**c**,**d**) catalysts.
